# Interpretable Radiomic Signature for Breast Microcalcification Detection and Classification

**DOI:** 10.1007/s10278-024-01012-1

**Published:** 2024-02-13

**Authors:** Francesco Prinzi, Alessia Orlando, Salvatore Gaglio, Salvatore Vitabile

**Affiliations:** 1 Department of Biomedicine, Neuroscience and Advanced Diagnostics (BiND), University of Palermo, Palermo, Italy; 2 Department of Computer Science and Technology, University of Cambridge, CB2 1TN Cambridge, United Kingdom; 3 Section of Radiology - Department of Biomedicine, Neuroscience and Advanced Diagnostics (BiND), University Hospital “Paolo Giaccone”, Palermo, Italy; 4 Department of Engineering, University of Palermo, Palermo, Italy; 5 Institute for High-Performance Computing and Networking, National Research Council (ICAR-CNR), Palermo, Italy

**Keywords:** Radiomics, Breast microcalcification, Machine learning, Interpretable signature

## Abstract

Breast microcalcifications are observed in 80% of mammograms, and a notable proportion can lead to invasive tumors. However, diagnosing microcalcifications is a highly complicated and error-prone process due to their diverse sizes, shapes, and subtle variations. In this study, we propose a radiomic signature that effectively differentiates between healthy tissue, benign microcalcifications, and malignant microcalcifications. Radiomic features were extracted from a proprietary dataset, composed of 380 healthy tissue, 136 benign, and 242 malignant microcalcifications ROIs. Subsequently, two distinct signatures were selected to differentiate between healthy tissue and microcalcifications (detection task) and between benign and malignant microcalcifications (classification task). Machine learning models, namely Support Vector Machine, Random Forest, and XGBoost, were employed as classifiers. The shared signature selected for both tasks was then used to train a multi-class model capable of simultaneously classifying healthy, benign, and malignant ROIs. A significant overlap was discovered between the detection and classification signatures. The performance of the models was highly promising, with XGBoost exhibiting an AUC-ROC of 0.830, 0.856, and 0.876 for healthy, benign, and malignant microcalcifications classification, respectively. The intrinsic interpretability of radiomic features, and the use of the Mean Score Decrease method for model introspection, enabled models’ clinical validation. In fact, the most important features, namely GLCM Contrast, FO Minimum and FO Entropy, were compared and found important in other studies on breast cancer.

**Supplementary Information:**

The online version contains supplementary material available at 10.1007/s10278-024-01012-1.

## Introduction

Breast cancer poses the greatest threat to women’s health and stands as the most prevalent malignancy globally. According to Sung et al. [1], over two million cases were diagnosed in 2020, making it the most frequently diagnosed cancer worldwide. The World Health Organization (WHO) [2] estimates indicate that female breast cancer has now surpassed lung cancer as the most commonly diagnosed form of cancer. Furthermore, the presence of breast microcalcifications is strongly linked to the risk of developing breast cancer. When microcalcifications and breast density are combined, they significantly amplify the risk of breast cancer, particularly in cases with higher levels of breast density. Breast calcifications are small deposits of calcium salts, with a diameter less than 1 mm [3], radio-opaque on mammograms. While they are quite common and mostly benign, breast calcifications serve as one of the earliest indicators of breast cancer on mammograms. Kim et al. [4] showed that in women with microcalcifications, the average time of breast cancer diagnosis was 7.9$\pm$ 1.8 years, whereas, in women without microcalcifications, the average time of breast cancer diagnosis was 8.5 ± 1.8 years. They can be detected in around one-third of all malignant lesions diagnosed during screening mammography [5, 6]. About 50% of non-palpable breast cancers and approximately 95% of all ductal carcinoma-in-situ (DCIS) are detected by mammography exclusively through microcalcification patterns [7, 8]. Furthermore, in a comprehensive meta-analysis conducted by Brennan et al. [9], it was found that while other mammographic abnormalities such as mass, architectural distortion and asymmetry, palpability of the lesion, and lesion size were strongly correlated with the upstaging of DCIS, DCIS manifesting as pure calcifications can also occult invasive disease. Breast microcalcifications classification may vary according to their size, shape, extent, density, and pattern of distribution on mammograms [10]. In clinical practice, their biopsy referral is based on radiologists’ assessment of the morphology and distribution according to the Breast Imaging-Reporting and Data System (BI-RADS) Atlas [11]. Nevertheless, false-positive biopsy rates for calcifications range from 30% to 87% [12, 13]. In addition, their localization becomes more complicated in low-contrast mammographic images and dense breast tissues [14]. In fact, the screening sensitivity for detecting malignant calcifications remains relatively low.

Various imaging modalities have been used to facilitate the diagnostic process, and machine learning methods have proved invaluable in this context [15, 16]. For instance, mammography serves as a standard screening tool for detecting specific abnormalities [17]. In such cases, several object detector architectures, including Yolo and Faster-RCNN, are employed for breast cancer localization and detection [18]. However, mammography may yield suboptimal results in cases of high breast density. Consequently, ultrasound plays a pivotal role in breast cancer diagnosis, serving both as a supplementary modality alongside mammography and as a primary imaging technique in certain regions [19]. In fact, machine learning-based tasks involving ultrasound images, such as segmentation [20] and classification [21], have gained prominence. Other examination modalities, like MRI, offer richer information for characterization purposes and are thus considered as an advanced examination [22]. In such instances, convolutional-based methods, Vision Transformers, and Radiomic techniques have seen widespread adoption [23]. However, the national prevention program recommends mammography as the primary screening examination, making it the main tool for early diagnosis of breast cancer. Screening sensitivity for the detection of malignant calcifications is low. Many detectable calcifications are not immediately flagged for further investigation but are instead identified during subsequent screening rounds when the disease has already progressed to an invasive stage. [24]. To mitigate this scenario, it is possible to enhance the physician’s diagnostic process by incorporating a quantitative perspective.

Radiomics is a new multidisciplinary approach that aims to convert images into meaningful data and informative biomarkers [25, 26]. Through radiomics it is possible to convert regions of interest (ROIs) into quantitative features to correlate a clinical outcome. In fact, after feature extraction, pre-processing, and selection, machine learning algorithms are used for model training and prediction. Radiomic feature extraction is also called *hand-crafted* features extraction: features are calculated through appropriate mathematical formulas applied to the gray levels histogram, to texture-defining matrices, or to the ROIs shape. Radiomic feature extraction has two enormous strengths. It is possible to extract radiomic features from ROIs at the original spatial resolution, avoiding any image resizing as is the case of deep feature extraction (e.g., via neural networks). Especially in the case of microcalcifications, in which the ROIs size is about 1 mm [3](e.g., a few pixels), the scaling can greatly reduce the information content. Moreover, since it is well known the meaning each radiomic feature expresses, it is possible to interpret the machine learning models’ findings and draw important clinical conclusions. This interpretation is a primary requirement to trust and validate the trained systems [27, 28].

**Fig. 1:**
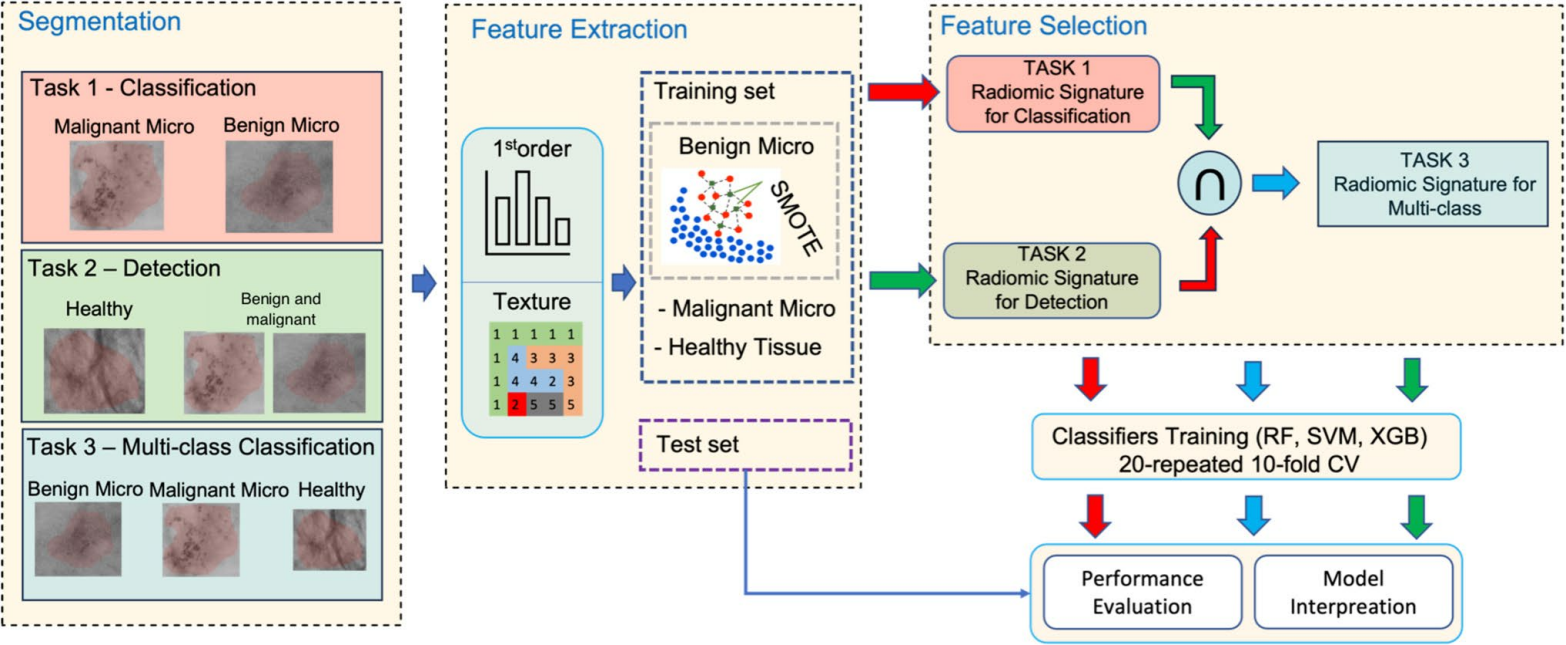
Overall architecture. The segmented data were divided into healthy tissue and benign and malignant microcalcifications. The same training pipeline was applied for task 1 (malignant vs. benign microcalcifications) and task 2 (healthy tissue vs. microcalcifications). In particular, after the feature extraction process, SMOTE was applied to the benign microcalcification samples for data balancing. Several feature selection steps were employed to select the best signature for tasks 1 and 2. The intersection between the two signatures was used to train a multi-class model, which can simultaneously distinguish healthy tissue, and benign and malignant microcalcifications (task 3). The validation performance were computed using a 20-repeated 10-fold cross-validation strategy. Finally, the performance of the trained models were computed on the test set, and their introspection was performed

The radiomic workflow has been applied in several medical contexts: to predict the involvement of lungs in COVID-19 and pneumonia using CT [29]; to predict myocardial function improvement in cardiac MR images in patients after coronary artery bypass grafting [30]; for molecular subtype classification of low-grade gliomas in MR imaging [31]; in breast cancer for predicting prognostic biomarkers and molecular subtypes in MRI[32], to predict axillary lymph node status [33], to predict the nodal status in ultrasound considering clinically negative breast cancer patients [34]; and for many other applications [35–40].

Also for microcalcification the radiomic workflow has been exploited. Lei et al. [41] used radiomics to predict benign BI-RADS 4 calcifications. They built a nomogram incorporating radiomic features and the menopausal state. Also Stelzer et al. [42] focused on Bi-Rads 4 microcalcification classification. Marathe et al. [43] presented a quantitative approach to classify benign and actionable (high-risk and malignant) amorphous calcifications. Loizidou et al. [44] acquired a proprietary dataset considering two sequential screening mammogram rounds. They exploited the temporal subtraction between the recent and prior mammograms, to classify between healthy tissue vs. microcalcification and benign vs. suspicious microcalcification. In Fanizzi et al. [45] radiomic and wavelet features were used for both normal vs. abnormal and benign vs. malignant classification.

As shown, it is common to divide the microcalcification analysis into two separate tasks: detection and classification. The detection aims to distinguish microcalcifications from healthy tissue. For classification instead, microcalcifications are assumed already been detected, and classification consists of distinguishing between malignant and benign. The small size of microcalcifications makes the detection process very sensitive because affected by factors such as human perception, breast density, and the nature of cancer itself [46]. For this reason, the capacity of radiomic workflow to provide a quantitative perspective, in addition to the visual assessment of physicians, can effectively support and enhance the diagnostic process.

In this work, a radiomic signature was proposed to train machine learning models for breast microcalcification detection and classification. In particular, a proprietary dataset collected at the Radiology section of University Hospital "Paolo Giaccone" (Palermo, Italy) was considered. Support Vector Machine (SVM), Random Forest (RF) and XGBoost (XGB) were compared both for detection and classification tasks. In addition, an analysis of the selected radiomic signature for the two tasks was performed to evaluate a common subset of radiomic features for simultaneous detection and classification. Indeed, we propose a radiomic signature able to distinguish between healthy tissue and benign and malignant microcalcifications. Figure 1 shows the general workflow. The main contributions of this study are:

- a well-structured processing pipeline [47] to define an informative radiomic signature for breast calcification;
- a multi-class model able to distinguish healthy tissue, benign and malignant microcalcifications;
- an interpretation of the more informative radiomic features to provide a trusted system supporting the decision-making processes.

This manuscript is structured as follows: "Materials and Methods" section describes the dataset, the extracted features, and the pipeline for machine learning model training; "Results" section reports the selected features and the performance for the detection (healthy vs. microcalcification), classification (benign vs. malignant microcalcification) and considering all the three classes; finally, "Discussion" and "Conclusions and Future Directions" sections conclude the paper, remarking the experimental findings and discussing the achieved results.

## Materials and Methods

The methodology used in this work includes two main macro topics: radiomics for feature extraction and shallow learning methods for training data-driven models. This architectural choice derives from several crucial aspects that must respect the models in clinical contexts: training with small dataset, highly accurate models, and explainable models [48]. The combination of shallow learning and radiomics meets all these requirements for the following reasons:

- **Radiomic Feature Extraction**: radiomics is concerned with the extraction of highly informative features for regions of interest characterization. Radiomic features are defined and standardized through the Imaging Biomarker Standardization Initiative (IBSI) and for this reason, allow reproducibility and comparison between different works. Effective and efficient extraction does not require training of deep learning models, but only the mask on which statistics and texture have to be calculated [49]. Moreover, the meaning expressed by each feature is well known (intelligible features), making it possible to study the features and correlate the meaning with already established clinical findings.
- **Highly Accurate Model**: the use of radiomic features transforms an image dataset into a tabular dataset, enabling the use of shallow learning models. It is well-established that shallow architectures demand a smaller volume of training data compared to deep architectures. As shown in [50] shallow learning methods like SVM outperform their deep learning counterparts when tabular data are used. In addition, SL approaches offer relatively straightforward interpretations, making them attractive for many applications in healthcare. As shown in [51] there are important technical and social reasons to prefer inherently intelligible AI models over deep neural models.
- **Interpretable Models**: shallow learning and explainable methods provide insights into the features driving their decisions, allowing clinicians to understand the reasoning behind the system’s recommendations. The union of explainable AI methods for the global explanation, shallow learning algorithms and radiomic features maintains an advantage by providing high-performance and highly interpretable models [52].

### Dataset Description and Segmentation

**Fig. 2:**
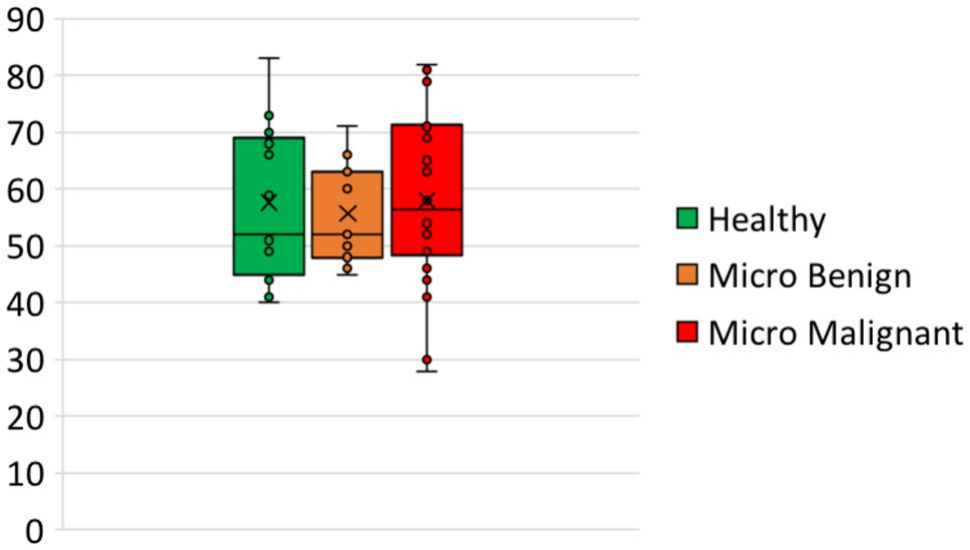
Patients age comparison among the three groups

A total of 161 images were acquired by a Fujifilm Full Field Digital Mammography at the Radiology section of the University Hospital "Paolo Giaccone" (Palermo, Italy). The images have a spatial resolution of 4728 × 5928 and a pixel size of 50 µm. The images were divided into healthy (76), benign microcalcifications (26), and malignant microcalcifications (59). The mean age is $57.6 \pm 12.7$ with a range of $40-83$ for the healthy patients, $55.7 \pm 8.6$ with a range of $45-71$ for benign microcalcification patients, $58.0 \pm 14.4$ with a range of $28-82$ for malignant microcalcification patients. Figure 2 compares the age box plots.

The ITK-SNAP toolkit was used for ROIs segmentation. The healthy ROIs were randomly selected and then manually segmented. For the microcalcification images instead, manual segmentation was performed to identify neighboring clusters of microcalcifications. Finally, 380 segmentations of healthy tissue, 136 benign and 242 malignant microcalcifications were collected. The annotations were performed by an expert radiologist dealing with the identification of abnormal regions. The first task, e.g. the detection task, was modeled considering the benign and malignant microcalcification vs. the healthy tissue (378 vs. 380 samples). The second task, e.g. the classification task, was performed considering the benign vs. the malignant microcalcifications (136 vs. 242). The third task, e.g. the multi-class classification task, was performed considering the benign vs. malignant microcalcifications vs. healthy tissue (136 vs. 242 vs. 380 samples).

### Radiomic Feature Extraction

In this work, we conformed to the standardization process in line with the IBSI [53] to ensure that the extracted features adhered to the required standards. To achieve this, the PyRadiomics library was used (version 3.0.1) [54], which is designed to be fully IBSI compliant. Ninety-three radiomic features were extracted, listed and discussed in "Radiomic Features" Section of the Supplementary Materials.

A bin-width of 25 was considered for image gray levels discretization. Considering the average range of 5419 (e.g., the difference between the maximum and minimum gray levels), this bin-width allows for about 216 bins histogram ($\frac{mean-range}{bin-width}$). Values of about 256 bins are commonly adopted [55].

The extracted features belong to intensity (or first-order (FO)) and textural features. First-order features define the intensity distribution of the pixel in a specified ROI. The texture features were computed from the following matrices: Gray Level Co-occurrence Matrix (GLCM) [56], Gray Level Run Length Matrix (GLRLM) [57–59], Neighboring Gray Tone Difference Matrix (NGTDM) [60], Gray Level Size Zone Matrix (GLSZM) [61] and Gray Level Dependence Matrix (GLDM) [62].

**Fig. 3:**
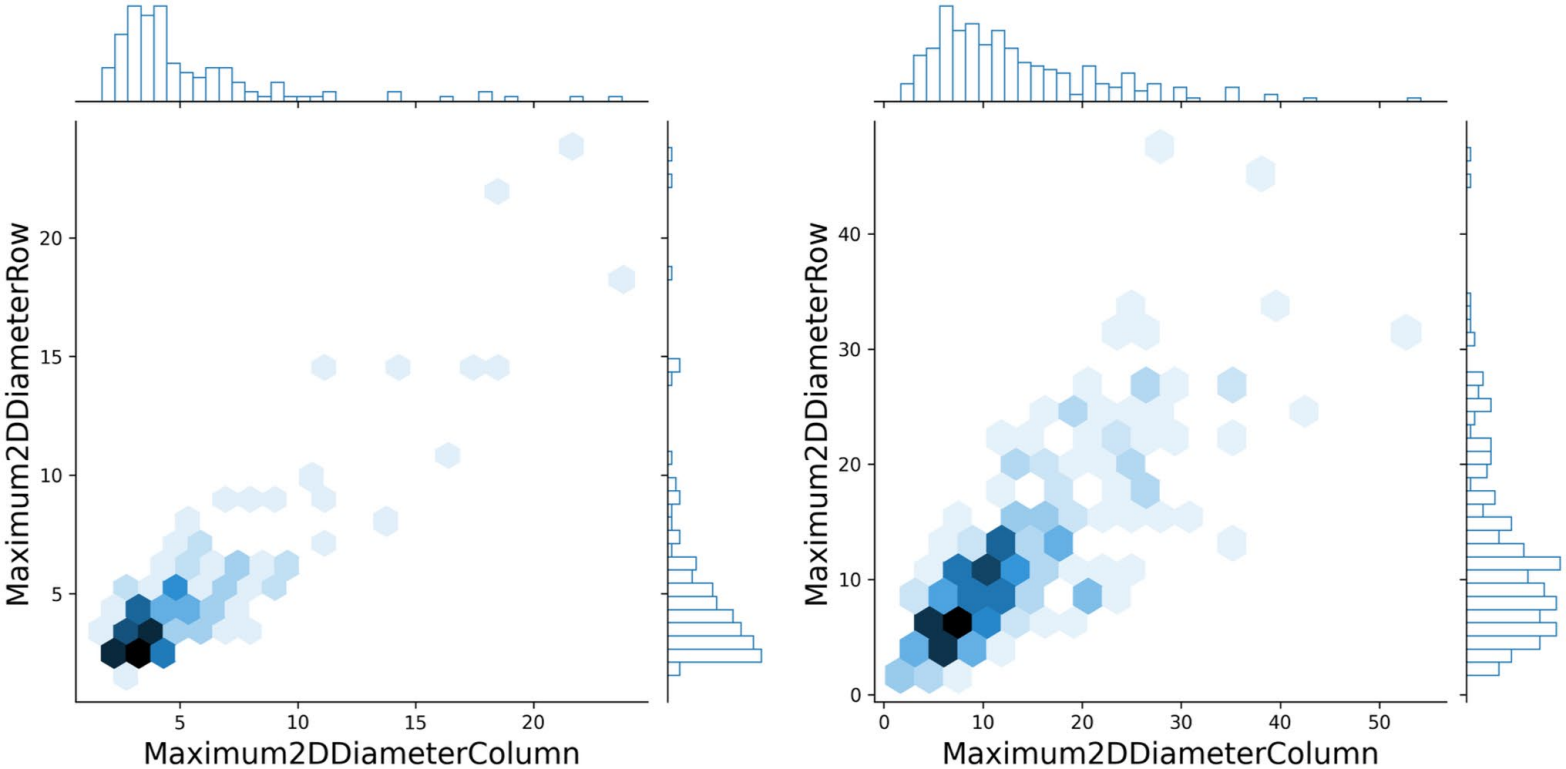
Microcalcifications size representation. Maximum 2D diameter Row (Column) is defined as the largest pairwise Euclidean distance between tumor surface mesh vertices in the column-slice (row-slice). These magnitudes represent the size width and height of lesions for benign (left image) and malignant (right image) microcalcifications

Instead, the 2D Shape features were not considered for the following reasons:

- To develop a signature independent of the generated segmentation, but dependent on texture and/or gray level intensity.
- As shown in Fig. 3left and right, the generated segmentations of malignant microcalcifications are on average larger than the benign ones. For this reason, shape features could introduce a major bias for the models, and discriminate only by shape and not by texture and/or gray level intensity.
- Finally, the segmentations are coarse because the work aims to detect and classify clusters and not individual microcalcifications.

### Feature Selection

In order to mitigate the risk of overfitting, several steps were executed in this study to reduce the initial feature set. In fact, the literature offers various relationships that define the appropriate number of features a model should incorporate based on the available training samples. From a purely statistical perspective, especially in the context of a binary classification problem, it is advisable to have around 10 to 15 samples for each feature incorporated into the radiomic signature [63]. This implies that a radiomic signature containing five features would require a dataset comprising between 50 and 75 patients for effective model training [47]. Exploiting this relationship, in the worst scenario of small dataset (task 2), 378 samples (242 malignant and 136 benign) allow for a 25-feature signature.

Two different signatures were selected for detection and classification tasks separately. In particular, variance analysis, correlation analysis, and statistical significance were performed to select an informative and non-redundant subset of radiomic features [47, 64]. All the near-constant features were discarded, considering a variance threshold of 0.01. The Spearman’s rank correlation coefficient was used to remove correlated features, considering $0.85$ as threshold [65, 66]. The Mann–Whitney U test was used to test the class differences (healthy tissue vs. microcalcifications and benign vs. malignant microcalcifications). A $p < 0.05$ was considered statistically significant.

Finally, the Sequential Forward Floating Selection (SFFS) algorithm was used [67] to select the best features subset for each model considered (e.g., RF, SVM, XGB). SFFS was applied for detection and classification tasks separately. In particular, the remaining features after analysis of variance, correlation, and statistical significance, were fed as input to SFFS. The models considered for SFFS were trained using a stratified 10-fold cross-validation strategy. Accuracy was the metric to maximize.

To train the multi-class model (e.g. simultaneously detection and classification), the common subset found for the two tasks separately was considered.

### Imbalanced Dataset Management

Considering the class imbalance between benign and malignant microcalcifications (Task 1) several strategies were implemented and compared. The Synthetic Minority Oversampling Technique (SMOTE) [68] is the most widely used technique for oversampling the minority class. In addition, ADASYN [69], BorderlineSMOTE [70] and KMeansSMOTE [71] were implemented. SMOTE-based methods are applied in countless works [72, 73], and its use is increasingly common [74].

The SMOTE-based techniques were applied to the training set to balance the two classes. Then, the minority class was over-sampled (i.e. the benign class) adding synthetic data to equalize the majority class (i.e. malignant class). No SMOTE was applied to the test set. This comparison was carried out before the training process, using the performance computed via SFFS and the three shallow learning algorithms employed in the work.

### Model Training and Test

Accurate extraction of radiomic features demonstrates its effectiveness in scenarios with limited data, in contrast to the data-intensive nature of deep training [49]. Additionally, radiomic features provide a valuable opportunity for leveraging shallow training methods with tabular data. In fact in this study, three different classifiers were implemented: SVM, RF, and XGB. RF and XGB are two widely employed Tree Ensemble algorithms. XGB aims to minimize the model’s loss function by incorporating weak learners through gradient descent, employing the Boosting Ensemble Method. On the other hand, RF employs the bagging technique to construct multiple weak learners by considering random subsets of features and bootstrap sample data. The decision of each learner is then aggregated using the Bagging Ensemble Method. Tree ensemble algorithms have demonstrated their effectiveness in classifying small datasets [75–77], making them among the most commonly employed alongside SVM [78]. Feature selection and model training were performed separately for detection and classification tasks. For this reason, it is possible to consider both tasks as binary classifications.

Before the feature selection and training stages, for the three tasks, the dataset was divided into 80% for feature selection and training, and the remaining 20% was used only for test. The test set was maintained separate from the tuning process, reserved solely for test (e.g., internal model validation [79]). In similar or smaller dataset-size the k-fold is typically used [80–82], while the Leave-One-Out (LOO) method is typically suggested in very-small dataset [83–85]. In addition, LOO validation is more susceptible to overfitting than k-fold cross-validation [55]. In any case, both k-fold cross-validation and leave-one-out cross-validation strategies were conducted. The k-fold was stratified and repeated 20 times. For this reason, the validation performance were reported considering the mean and standard deviation for each metric. The model that exhibited the highest accuracy during the validation phase was selected for testing. The features that overlapped between the selected ones for the detection and classification tasks were used to train the multi-class model, employing the same training and testing procedure.

To evaluate model performance, Accuracy, Area Under the Receiver Operating Characteristic (AUC-ROC), Specificity, Sensitivity, Positive Predictive Value (PPV), Negative Predictive Value (NPV)  and F-Score were considered. In addition, to ensure an accurate comparison between the trained models, the same seed was set for all probabilistic terms in the algorithms and for the splits generation for the stratified cross-validation.

## Results

The experiments were conducted in Python 3.7 environment. RF was trained using the bootstrap technique, 100 estimators and the Gini criterion; XGB was trained using 100 estimators, 6 as max depth, ‘gain’ as importance type, binary logistic as loss function and 0.3 as learning rate. SVM was trained using the Radial basis kernel, regularization parameter $C=1.0$ and kernel coefficient to $1 / (n_{features} * variance)$. For SVM, features were standardized before the training.

In addition, for multi-class training, the one-vs-rest strategy for SVM and the softmax loss function for XGB were used.

### Features Selected and SMOTE Evaluation

Table 1 shows the selected features for the two tasks after the application of the variance analysis, correlation analysis, and statistical test. In particular, an important overlapping was found between the two subsets. Then, the SFFS method was applied for SMOTE-based data balancing comparison and for selecting the best signature for the classification and detection tasks.

**Table 1:** Selected features for detection and classification tasks, before applying SFFS

Feature	Class	Det	Clas
10Percentile	FO	X	X
90Percentile	FO	X	X
Energy	FO		X
Entropy	FO	X	X
Kurtosis	FO		X
Maximum	FO	X	X
Minimum	FO	X	X
Skewness	FO	X	X
Autocorrelation	GLCM		X
Contrast	GLCM	X	X
DependenceVariance	GLDM		X
LargeAreaLowGrayLevelEmphasis	GLSZM	X	X
Busyness	NGTDM		X
Contrast	NGTDM	X	

Table 2 shows the accuracy values found by SFFS considering the subset maximizes the accuracy. In particular, no significant differences were found between the implemented methods, with SMOTE providing slightly higher performance. Therefore, SMOTE was eventually selected as the data balancing method.

**Table 2:** Comparison of class balancing methods in terms of accuracy

Method	XGB	RF	SVM
SMOTE	0.897 ± 0.050	0.894 ± 0.042	0.864 ± 0.041
ADASYN	0.891 ± 0.066	0.888 ± 0.031	0.852 ± 0.031
BorderlineSMOTE	0.892 ± 0.056	0.889 ± 0.047	0.864 ± 0.037
KMeansSMOTE	0.892 ± 0.055	0.889 ± 0.047	0.863 ± 0.037

**Fig. 4:**
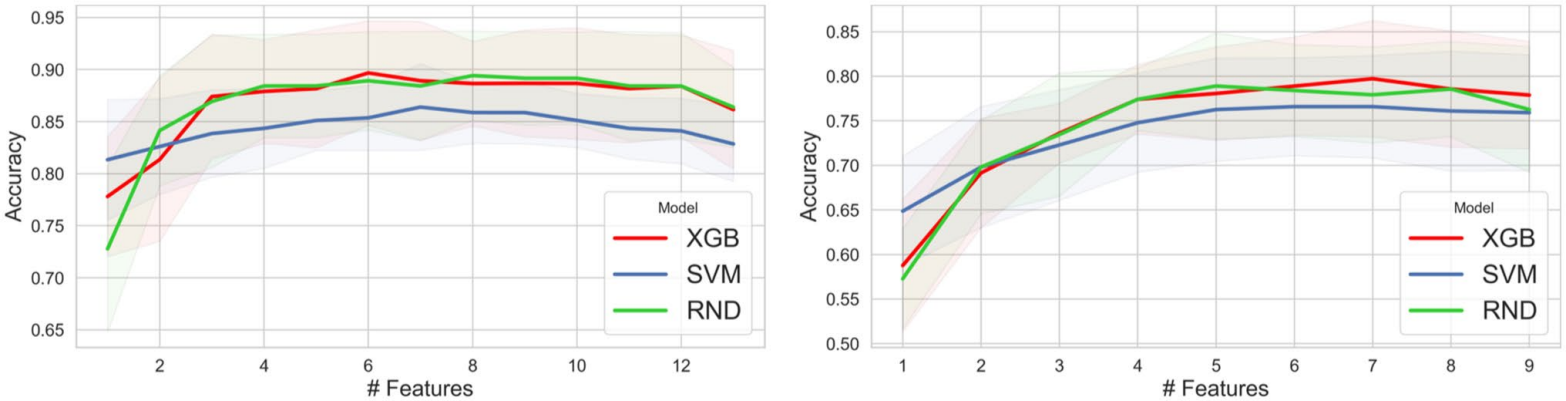
The graph generated via SFFS shows the accuracy value for each model (XGB, SVM, and RND) considering several feature subsets. The x-axis represents the $n-th$ step of the algorithm; the y-axis instead shows the accuracy value. On average, 7 is the features number that maximizes the accuracy of the three models for detection (left image) and classification (right image) tasks

For detection and classification tasks, each model (e.g., SVM, XGB, RF) was trained considering the same number of features, computed via SFFS by considering the smallest radiomic signature providing the highest accuracy. In particular, Fig. 4left and right show the calculated accuracy considering the different subsets selected via SFFS for detection and classification tasks, respectively. Figure 4left illustrates that, on average, a signature size of seven maximizes accuracy for all three models in the detection task. Figure 4right demonstrates that a set of seven features also optimizes the classification task accuracy. For this reason, seven features were selected for detection and classification task training. For the detection task, the NGTDM Contrast feature was the first one selected via SFFS for each considered model. The NGTDM Contrast was not statistically significant for the classification task. The FO Entropy feature was the first selected via SFFS in the classification task for each considered model. In addition, FO Entropy, GLCM Contrast and GLSZM LargeAreaLowGrayLevelEmphasis were the most frequently selected features via SFFS, that is, in at least 5 of the 6 models considered (RF, SVM and XGB for detection; RF, SVM and XGB for classification).

Considering the overlap between the features statistically significant ($p < 0.05$) for the detection and classification task, the common subset was used to solve the two tasks simultaneously. Specifically, the 8 common features, shown in Table 1, were used to solve a multi-class problem, considering three classes: healthy tissue, and benign and malignant microcalcifications. For this reason, the results section is organized to expose the results of the three tasks separately.

### Performance of the Three Tasks

**Fig. 5:**
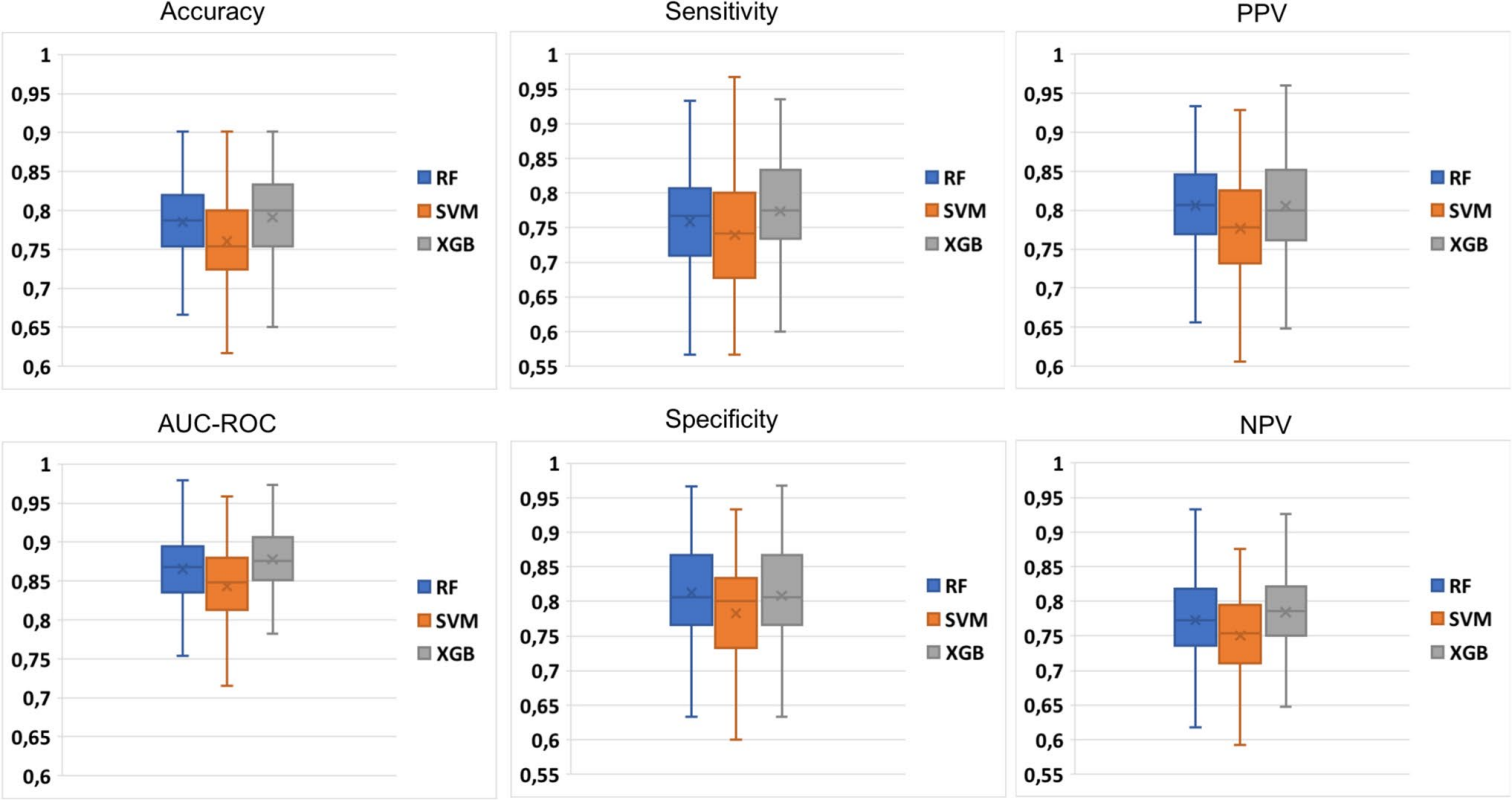
Validation performance for the detection task computed during the 20-repeated 10-fold cross-validation procedure

**Fig. 6:**
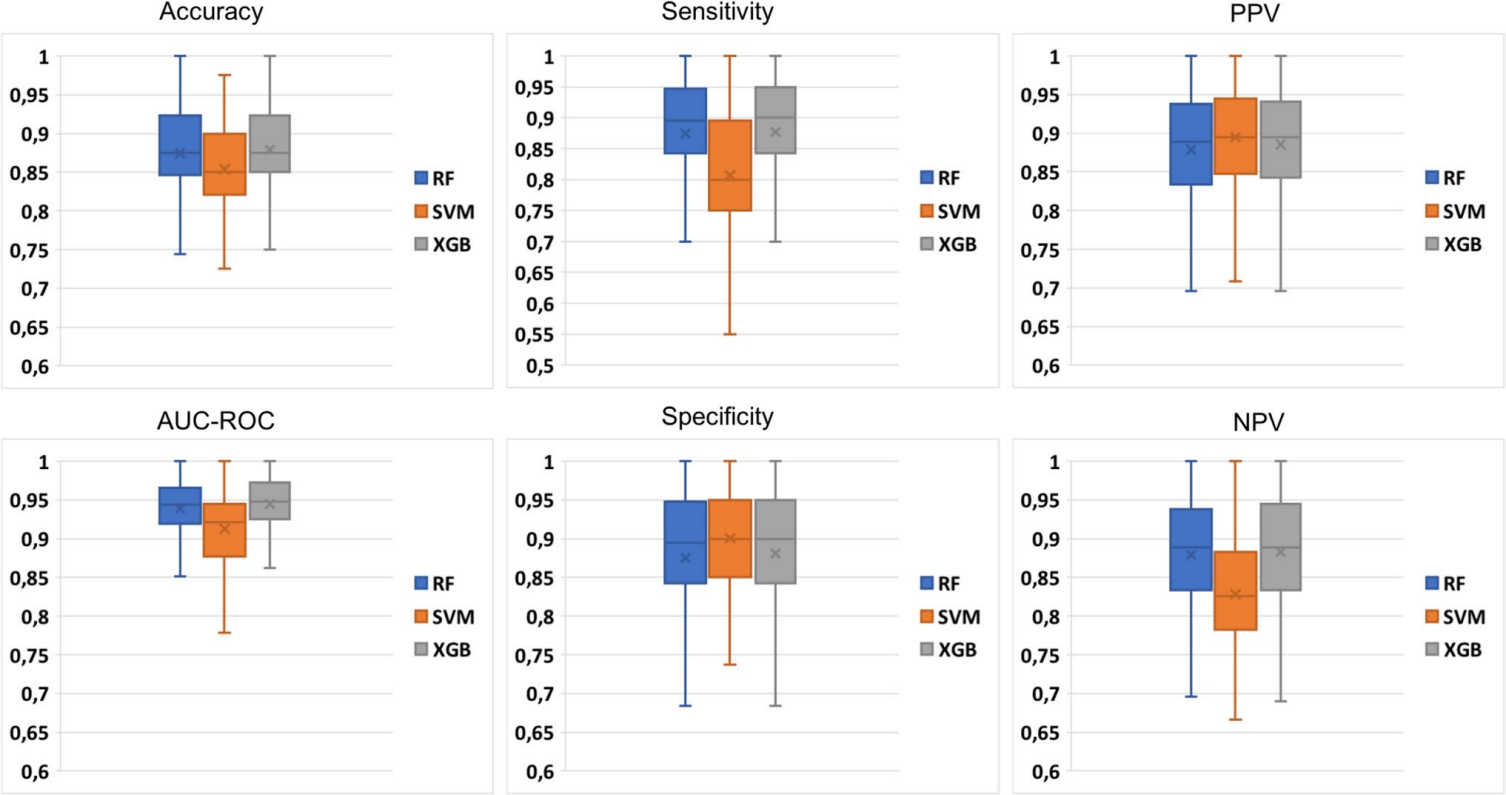
Validation performance for the classification task computed during the 20-repeated 10-fold cross-validation procedure

The performance evaluation during feature selection via SFFS was conducted using a 10-fold stratified cross-validation approach (refer to Fig. 4left and right). The cross-validation process was repeated only once due to the computational complexity of the SFFS algorithm. Conversely, for model validation, a 10-fold cross-validation was repeated 20 times to ensure a more accurate evaluation of the models (refer to Figs. 5 and 6). The LOO performance are reported in Section "Leave-One-Out performance" of Supplementary Material. Ultimately, the most accurate model determined in the validation phase, was selected for testing on the independent test dataset (refer to Tables 3, 4 and 5).

#### Detection Performance

This task aims to classify healthy tissue from microcalcification. The training set consisted of 306 healthy tissue samples and 302 microcalcifications; the test set of 78 microcalcifications and 74 healthy tissues. Figure 5 shows the validation performance computed during the 20-repeated 10-fold cross-validation. The performance in Fig. 5 are comparable with the one in LOO, shown in Table 7 of Supplementary Materials. XGB achieved a higher performance in the validation phase, almost comparable with RF. For each model, a higher specificity was found with respect to the sensitivity. It means a higher capability of the models to recognize the healthy tissue rather than microcalcifications.

In Table 3 are shown the metrics computed in the test phase. While SVM exhibited lower performance compared to XGB and RF during the validation phase, it demonstrated superior generalization capabilities when applied to unseen data. In particular, SVM achieved an AUC-ROC of 0.865. Also, RF and XGB reached promising AUC-ROC performance of 0.859 and 0.854 respectively. However, a strong imbalance between sensitivity and specificity was computed, with a higher specificity than sensitivity.

**Table 3:** Test performance for the detection task

Metric	RF	SVM	XGB
Accuracy	0.756	0.789	0.750
AUC-ROC	0.859	0.865	0.854
Sensitivity	0.729	0.783	0.702
Specificity	0.782	0.794	0.794
PPV	0.760	0.783	0.764
NPV	0.753	0.794	0.738
F-Score	0.744	0.783	0.720

**Table 4:** Test performance for the classification task

Metric	RF	SVM	XGB
Accuracy	0.868	0.868	0.842
AUC-ROC	0.921	0.927	0.933
Sensitivity	0.931	0.863	0.909
Specificity	0.781	0.875	0.750
PPV	0.854	0.904	0.833
NPV	0.892	0.823	0.857
F-Score	0.891	0.883	0.870

#### Classification Performance

This task aims to classify the benign and the malignant microcalcifications. The training set consisted of 198 malignant microcalcifications and 198 benign microcalcifications (considering 104 real samples and 95 synthetic samples generated via SMOTE). The test set consisted of 44 malignant and 32 benign microcalcifications. Figure 6 shows the validation performance. The performance in Fig. 6 are comparable with the one in LOO, shown in Table 8 of Supplementary Materials. The achieved performance in the test phase were reported in Table 4.

As the detection task, SVM exhibited lower performance compared to XGB and RF during the validation phase. However in the test phase decision tree-based models perform poorer than SVM, and again with a strong imbalance between sensitivity and specificity. However, the models result in very high performance, with an AUC-ROC of 0.921, 0.927 and 0.933 for RF, SVM and XGB, respectively. For decision tree-based models, a higher sensitivity was computed with respect to specificity. It means a higher capability of the models to recognize malignant rather than benign microcalcifications.

#### Multi-class Model Performance

Considering the overlap between the discriminating features for the detection and classification tasks (Table 1), the common features set was used to address the two tasks simultaneously. For this reason, SVM, RF and XGB were trained for multi-class classification, considering the one-vs-rest strategy for SVM and the softmax loss function for XGB. In this case, 198 malignant microcalcifications, 198 benign microcalcifications (104 real and 97 generated via SMOTE), and 198 healthy samples were considered for the training set. The 198 healthy samples were randomly selected from the original 380 to avoid class imbalance in training. For the test instead, 78, 44 and 32 were used for healthy, benign and malignant microcalcification, respectively.

Table 5 shows the achieved test performance. For healthy tissue, a high specificity and a low sensitivity were computed. This means that the model is more capable of detecting microcalcification than healthy tissue. A similar observation applies to benign microcalcifications, wherein the model finds it easier to detect both malignant microcalcifications and healthy tissue. Consequently, in each scenario, the detection of malignant microcalcifications is comparatively more straightforward. For this task, the decision tree-based models outperform the SVM classifiers, obtaining a higher AUC-ROC and accuracy for the recognition of the three classes. This means that tree-based models are more appropriate for multi-class classification.

**Table 5:** Multi-class classification test performance, for simultaneous detection and classification task

Model	Class	Acc	AUC	Sens	Spec	PPV	NPV	F-Score
	Healthy	0.746	0.810	0.679	0.815	0.791	0.712	0.731
RF	B Micro	0.818	0.860	0.593	0.877	0.558	0.891	0.575
	M Micro	0.811	0.890	0.772	0.827	0.641	0.900	0.700
	Healthy	0.694	0.783	0.538	0.855	0.792	0.643	0.640
SVM	B Micro	0.792	0.849	0.687	0.819	0.500	0.909	0.578
	M Micro	0.824	0.840	0.818	0.649	0.927	0.882	0.870
	Healthy	0.740	0.830	0.679	0.802	0.779	0.709	0.725
XGB	B Micro	0.811	0.856	0.625	0.860	0.540	0.897	0.580
	M Micro	0.824	0.876	0.750	0.854	0.673	0.895	0.710

## Discussion

The work addressed the problem of breast microcalcifications to propose a data-driven system to support the physician’s diagnostic process. By using the radiomic workflow, the images were transformed into highly informative features, offering a quantitative perspective that complements the visual assessment of physicians. Considering the difficulty of microcalcifications diagnosis and their extremely small size, data-driven systems can play a crucial role. Indeed, a considerable proportion of microcalcifications progress into invasive lesions, underscoring the significance of early detection in preventing advanced stages of the disease and facilitating appropriate management. In this context, the radiomics workflow combined with the shallow learning techniques can support the physician’s diagnostic process, as well as enable feature interpretation and explainable models. Explainable models are crucial for model validation and to compare the findings with the medical literature [86]. In addition, explainability improves the usability and acceptability of AI models [27]. In many intensive decision-based tasks, the interpretability of an AI-based system may emerge as an indispensable feature [28]. In fact, our work presents important results, both in terms of predictive performance and findings resulting from the interpretability of radiomic features.

### Model Performance and Findings Interpretation

Focusing on performance, the detection performance were promising, showing an AUC-ROC of 0.859, 0.856 and 0.854 for RF, SVM and XGB, respectively. The performance increases when only microcalcifications are considered for malignant vs. benign classification, showing an AUC-ROC of 0.921, 0.927 and 0.933 for RF, SVM and XGB. This result is important because it means that the system is capable of detecting lesions that degenerate into invasive cancers. The difference in performance between the two tasks confirms that the main difficulty in the analysis of microcalcifications lies precisely in detection, which is the crucial task in screening for early diagnosis.

**Fig. 7:**
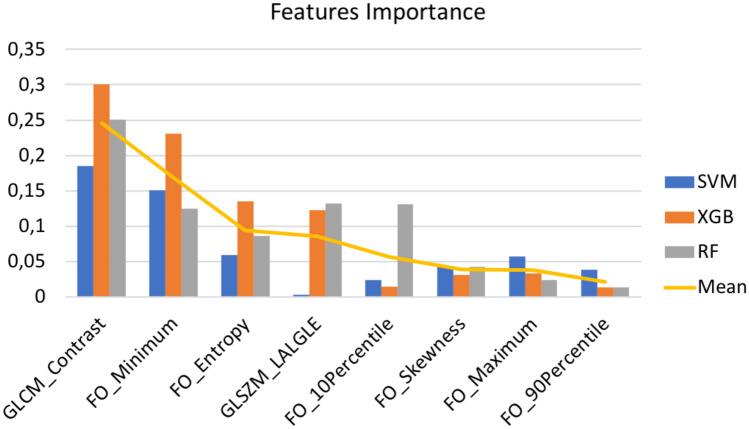
Features importance computed via the mean score decrease method

One of the main results lies in the discovery of an overlapping radiomic signature between the detection and classification tasks. In particular, Fig. 7 shows the importance of the features calculated using the Mean Decrease Accuracy method available in ELI5 framework [87]. The GLCM Contrast, FO Entropy, and FO Minimum represent the most important features. The GLCM Contrast is a measure of the local intensity variation, so a larger value correlates with a greater disparity in intensity values among neighboring pixels. We found a higher Contrast in healthy tissue with respect to microcalcification. A higher Minimum was found for the healthy tissue with respect to microcalcification: this is intuitive because the microcalcification intensity is much lower compared with healthy tissue. Finally, a higher Entropy was found in microcalcifications compared with the healthy tissue. With the Entropy is possible to measure the uncertainty/randomness in the image values. Unlike deep architectures, where feature extraction produces a latent space that lacks comparability and reproducibility with other works, radiomic workflow enables the comparison of significant features across different studies. This is achieved due to the known meaning associated with each feature, in contrast to deep features. Through this approach, a significant overlap was discovered with other studies. In fact, Entropy and Minimum were found important in PET and MRI for breast cancer phenotypes and prognosis [88]; again Entropy in multiparametric MRI for breast cancer tissue characterization [89, 90], and also the GLCM Contrast [89]; the Minimum in Dynamic Contrast-Enhanced MRI (DCE-MRI) for Sentinel Lymph Node Metastasis prediction [91].

#### Comparison

Several papers addressed the microcalcification analysis through radiomics. Although the following works use different datasets, a qualitative comparison was performed and shown in Table 6. In particular, Stelzer et al. [42] have focused only on BI-RADS 4 microcalcification, analyzing a dataset consisting of 150 benign and 76 malignant microcalcifications. They exploited the radiomic workflow for classification, in an attempt to avoid unnecessary benign biopsies. To the extracted features, the principal component analysis (PCA) was applied and a multilayer perceptron was trained. They obtained an AUC-ROC of 0.82$-$0.83 and found the GLCM Contrast the most important feature contributing to PCA. Lei et al. [41] focused also on BI-RADS 4 calcifications to discriminate benign from malignant calcifications. They selected 6 radiomic features and used the menopausal state to train an SVM model, reaching an AUC-ROC of 0.80, a PPV of 73.53, and NPV of 84.21. Marathe et al. [43] analyzed 276 amorphous calcifications (200 benign and 76 malignant). They extracted the radiomic features from the foreground and background masks, and global features from dilated foreground masks. Using the LightGBM classifier they obtained an AUC-ROC of 0.73, a sensitivity of 1.0 and a specificity of 0.35. In addition, they proved that in small dataset scenario, local and global radiomic features allows higher performance with respect to VGG-16 and ResNet-50 deep architecture. In Fanizzi et al. [45] the healthy ROIs were considered to train two different classifiers: normal vs. abnormal and benign vs. malignant. From the Breast Cancer Digital Repository [94] 130 microcalcifications (75 benign and 55 malignant) and 130 healthy ROIs were selected. They used the wavelet Haar transform before the feature extraction process. The selected features were used to train the random forest model, obtaining a median AUC-ROC value of 98.16% and 92.08% for the detection and classification tasks, respectively. As discussed, we found an opposite trend: the classification model performed better than the detection model. Loizidou et al. [44] acquired a proprietary dataset considering two sequential screening mammogram rounds, to distinguish between normal tissue vs. microcalcifications, and benign vs. suspicious microcalcifications. For the two tasks, radiomic features from the recent mammogram (RM) and from the temporal subtracted (TS) mammograms were extracted. Then, several machine learning classifiers were compared, considering the RM and TS selected signatures for the two tasks. Focusing on the RM modality, a lower sensitivity and higher specificity were computed for the detection task, as in our work. In addition, compared to our work, they obtained a higher accuracy but an AUC-ROC significantly lower. However, their performance increased significantly when the TS modality was considered. In Li et al. [92] a proprietary dataset composed by 260 patients with non-palpable microcalcifications and BI-RADS 4 was used to propose a signature to distinguish between noncancerous and cancerous microcalcifications. They used several higher-level radiomic features including Laplacian of Gaussian (LoG) spatial filters, single-level coiflet decomposition, and Local Binary Pattern (LBP). Then, several shallow learning algorithms were implemented, showing an AUC of 0.906 using SVM. Predicting invasion carcinoma from DCIS lesions diagnosed was investigated in [93]. Using 161 pure DCIS and 89 DCIS with invasion, radiomic and clinical features were used to train an XGB model, showing an AUROC of 0.72 (Table 6).

**Table 6:** Comparison with similar works

Work	Dataset Size	Lesion Type	Features	Methods	Performance (AUROC)
[42]	B:150, M:76	BI-RADS 4 MC	Radiomic	PCA, MLP	Classification: 0.82$-$0.83
[41]	a) 159 (49.06% M ratio) b) 53 (52.83% M ratio)	BI-RADS 4 MC	Radiomic, Menopausal state	LASSO, SVM	Classification: 0.80
[43]	B:200, M:76	Amorphous MC	Local: Radiomic Global: Topological structure features	LightGBM	Classification: 0.73
[45]	B:75, M:55, H:130	MC and H ROIs	Wavelet-derived statistical features	Embedded methods, filter methods, Random Forest	Detection: 98.16 Classification: 92.08
[44]	H:40, Suspicious:40	MC	Radiomic from RM and TS	Statistical test, SVM, TreeEnsemble	Using only RM: Detection: 0.77 Classification: 0.79
[92]	B:130, M:130	non-palpable MC with BI-RADS 4	Radiomic features, LoG-derived, Wavelet-derived, LBP-derived	ANOVA, RFE, SVM	Classification: 0.906
[93]	Pure DCIS:161, DCIS with invasion:89	Suspicious MC in DCIS	Radiomic, clinical imaging characteristics	Logistic Regression, XGB	Classification: 0.72
our	H: 380, M: 136, H: 242	MC	Radiomic	SFS, SVM, XGB, RF	Detection: 0.865, Classification: 0.933

*MC* microcalcification, *B* benign, *M* malignant, *H* healthy

## Conclusions and Future Directions

This work aimed to train a radiomic model for breast microcalcifications diagnosis. The signatures extracted for the detection and classification tasks were used also to train a multi-class model to distinguish healthy tissue, benign and malignant microcalcifications. The proposed signature introduces several quantitative biomarkers to support the diagnostic process. The performance appears promising and comparable or higher with the literature.

As emphasized by Caroprese et al. [95], following the explicit incorporation of the right to explanation within the General Data Protection Regulation, the urgent need for fully transparent and interpretable models has emerged. Our research is dedicated to enhancing model interpretability by introducing intelligible input in the form of radiomic features and employing a post-hoc explanation method. The fusion of these two elements renders the model comprehensible on a global scale, facilitating its clinical validation. However, it’s worth noting that we have not conducted an analysis for locally explaining the model, which is an important aspect for justifying and reinforcing the model’s results [28]. One of the most intriguing and promising developments in the field of breast cancer research and neural networks is the integration of histopathological images. With the advent of deep learning technologies, researchers are making significant strides in improving the accuracy and efficiency of breast cancer diagnosis and prognosis. It was shown for example the promising for invasive ductal carcinoma breast cancer grade classification using an ensemble of convolutional neural networks [96]. Convolutional-based neural networks showed promising results also on the classification of invasive and non‑invasive cancer [97]. Another avenue for further exploration is the relationship between intelligible features, such as radiomic features, and learned features extracted from neural networks. While radiomic features contribute to model explainability, deep features enhance model accuracy [98]. This study has the potential to delve into the well-known trade-off between explainability and accuracy, a subject of interest highly discussed [99, 100].

### Supplementary Information

Below is the link to the electronic supplementary material.

Supplementary file 1 (pdf 134 KB)

## Data Availability

Data will be made available on reasonable request.

## References

[1] Sung, H., Ferlay, J., Siegel, R.L., Laversanne, M., Soerjomataram, I., Jemal, A., Bray, F.: Global cancer statistics 2020: Globocan estimates of incidence and mortality worldwide for 36 cancers in 185 countries. CA: a cancer journal for clinicians **71**(3), 209–249 (2021) 10.3322/caac.21660 10.3322/caac.21660 33538338

[2] Organization, W.H., et al.: Global health estimates 2020: deaths by cause, age, sex, by country and by region, 2000–2019. WHO Geneva, Switzerland (2020)

[3] Azam S, Eriksson M, Sjölander A, Gabrielson M, Hellgren R, Czene K, Hall P (2021). Predictors of mammographic microcalcifications. International journal of cancer.

[4] Kim S, Tran TXM, Song H, Park B (2022). Microcalcifications, mammographic breast density, and risk of breast cancer: a cohort study. Breast Cancer Research.

[5] Azam S, Eriksson M, Sjölander A, Gabrielson M, Hellgren R, Czene K, Hall P (2021). Mammographic microcalcifications and risk of breast cancer. British journal of cancer.

[6] Muttarak M, Kongmebhol P, Sukhamwang N (2009). Breast calcifications: which are malignant. Singapore Med J.

[7] Scimeca, M., Bonfiglio, R., Menichini, E., Albonici, L., Urbano, N., De Caro, M.T., Mauriello, A., Schillaci, O., Gambacurta, A., Bonanno, E.: Microcalcifications drive breast cancer occurrence and development by macrophage-mediated epithelial to mesenchymal transition. International journal of molecular sciences **20**(22), 5633 (2019) 10.3390/ijms20225633 10.3390/ijms20225633 PMC6888678 31718020

[8] Narod SA (2011). Age of diagnosis, tumor size, and survival after breast cancer: implications for mammographic screening. Breast cancer research and treatment.

[9] Brennan ME, Turner RM, Ciatto S, Marinovich ML, French JR, Macaskill P, Houssami N (2011). Ductal carcinoma in situ at core-needle biopsy: meta-analysis of underestimation and predictors of invasive breast cancer. Radiology.

[10] Tot, T., Gere, M., Hofmeyer, S., Bauer, A., Pellas, U.: The clinical value of detecting microcalcifications on a mammogram. In: Seminars in Cancer Biology, vol. 72, pp. 165–174 (2021). 10.1016/j.semcancer.2019.10.024. Elsevier 10.1016/j.semcancer.2019.10.024 31733292

[11] American College of Radiology BI-RADS Committee: Acr Bi-rads Atlas: Breast Imaging Reporting and Data System, p. American College of Radiology

[12] Bent CK, Bassett LW, D’Orsi CJ, Sayre JW (2010). The positive predictive value of bi-rads microcalcification descriptors and final assessment categories. American Journal of Roentgenology.

[13] Grimm LJ, Miller MM, Thomas SM, Liu Y, Lo JY, Hwang ES, Hyslop T, Ryser MD (2019). Growth dynamics of mammographic calcifications: differentiating ductal carcinoma in situ from benign breast disease. Radiology.

[14] Salvado, J., Roque, B.: Detection of calcifications in digital mammograms using wavelet analysis and contrast enhancement. In: IEEE International Workshop on Intelligent Signal Processing, 2005., pp. 200–205 (2005). 10.1109/WISP.2005.1531658. IEEE

[15] Abhisheka, B., Biswas, S.K., Purkayastha, B.: A comprehensive review on breast cancer detection, classification and segmentation using deep learning. Archives of Computational Methods in Engineering, 1–30 (2023) 10.1007/s11831-023-09968-z

[16] Loizidou, K., Elia, R., Pitris, C.: Computer-aided breast cancer detection and classification in mammography: A comprehensive review. Computers in Biology and Medicine, 106554 (2023) 10.1016/j.compbiomed.2023.106554 10.1016/j.compbiomed.2023.106554 36646021

[17] Prinzi, F., Insalaco, M., Orlando, A., Gaglio, S., Vitabile, S.: A yolo-based model for breast cancer detection in mammograms. Cognitive Computation 16, 107–120 (2024) 10.1007/s12559-023-10189-6

[18] Galić I, Habijan M, Leventić H, Romić K (2023). Machine learning empowering personalized medicine: A comprehensive review of medical image analysis methods. Electronics.

[19] Vocaturo, E., Zumpano, E.: Artificial intelligence approaches on ultrasound for breast cancer diagnosis. In: 2021 IEEE International Conference on Bioinformatics and Biomedicine (BIBM), pp. 3116–3121 (2021). 10.1109/BIBM52615.2021.9669690. IEEE

[20] Wang, Z.: Deep learning in medical ultrasound image segmentation: a review. arXiv preprint arXiv:2002.07703 (2020)

[21] Shen Y, Shamout FE, Oliver JR, Witowski J, Kannan K, Park J, Wu N, Huddleston C, Wolfson S, Millet A (2021). Artificial intelligence system reduces false-positive findings in the interpretation of breast ultrasound exams. Nature communications.

[22] Militello C, Rundo L, Dimarco M, Orlando A, Woitek R, D’Angelo I, Russo G, Bartolotta TV (2022). 3d dce-mri radiomic analysis for malignant lesion prediction in breast cancer patients. Academic Radiology.

[23] Adam R, Dell’Aquila K, Hodges L, Maldjian T, Duong TQ (2023). Deep learning applications to breast cancer detection by magnetic resonance imaging: a literature review. Breast Cancer Research.

[24] Mordang J, Gubern-Mérida A, Bria A, Tortorella F, Mann R, Broeders M, Heeten G, Karssemeijer N (2018). The importance of early detection of calcifications associated with breast cancer in screening. Breast cancer research and treatment.

[25] Lambin P, Rios-Velazquez E, Leijenaar R, Carvalho S, Van Stiphout RG, Granton P, Zegers CM, Gillies R, Boellard R, Dekker A, Aerts HJ (2012). Radiomics: extracting more information from medical images using advanced feature analysis. European journal of cancer.

[26] Gillies RJ, Kinahan PE, Hricak H (2016). Radiomics: images are more than pictures, they are data. Radiology.

[27] Minh D, Wang HX, Li YF, Nguyen TN (2022). Explainable artificial intelligence: a comprehensive review. Artificial Intelligence Review.

[28] Combi, C., Amico, B., Bellazzi, R., Holzinger, A., Moore, J.H., Zitnik, M., Holmes, J.H.: A manifesto on explainability for artificial intelligence in medicine. Artificial Intelligence in Medicine **133**, 102423 (2022) 10.1016/j.artmed.2022.102423 10.1016/j.artmed.2022.102423 36328669

[29] Khaniabadi, P.M., Bouchareb, Y., Al-Dhuhli, H., Shiri, I., Al-Kindi, F., Khaniabadi, B.M., Zaidi, H., Rahmim, A.: Two-step machine learning to diagnose and predict involvement of lungs in covid-19 and pneumonia using ct radiomics. Computers in biology and medicine **150**, 106165 (2022) 10.1016/j.compbiomed.2022.106165 10.1016/j.compbiomed.2022.106165 PMC9533634 36215849

[30] Arian F, Amini M, Mostafaei S, Rezaei Kalantari K, Haddadi Avval A, Shahbazi Z, Kasani K, Bitarafan Rajabi A, Chatterjee S, Oveisi M, Shiri I, Zaidi H (2022). Myocardial function prediction after coronary artery bypass grafting using mri radiomic features and machine learning algorithms. Journal of digital imaging.

[31] Lam LHT, Do DT, Diep DTN, Nguyet DLN, Truong QD, Tri TT, Thanh HN, Le NQK (2022). Molecular subtype classification of low-grade gliomas using magnetic resonance imaging-based radiomics and machine learning. NMR in Biomedicine.

[32] Lee JY, Lee K-S, Seo BK, Cho KR, Woo OH, Song SE, Kim E-K, Lee HY, Kim JS, Cha J (2022). Radiomic machine learning for predicting prognostic biomarkers and molecular subtypes of breast cancer using tumor heterogeneity and angiogenesis properties on mri. European Radiology.

[33] Cheng J, Ren C, Liu G, Shui R, Zhang Y, Li J, Shao Z (2022). Development of high-resolution dedicated pet-based radiomics machine learning model to predict axillary lymph node status in early-stage breast cancer. Cancers.

[34] Bove S, Comes MC, Lorusso V, Cristofaro C, Didonna V, Gatta G, Giotta F, La Forgia D, Latorre A, Pastena MI, Petruzzellis N, Pomarico D, Rinaldi L, Tamborra P, Zito A, Fanizzi A, Massafra R (2022). A ultrasound-based radiomic approach to predict the nodal status in clinically negative breast cancer patients. Scientific Reports.

[35] Vicini, S., Bortolotto, C., Rengo, M., Ballerini, D., Bellini, D., Carbone, I., Preda, L., Laghi, A., Coppola, F., Faggioni, L.: A narrative review on current imaging applications of artificial intelligence and radiomics in oncology: focus on the three most common cancers. La radiologia medica, 1–18 (2022) 10.1007/s11547-022-01512-6 10.1007/s11547-022-01512-6 35771379

[36] Carlini, G., Gaudiano, C., Golfieri, R., Curti, N., Biondi, R., Bianchi, L., Schiavina, R., Giunchi, F., Faggioni, L., Giampieri, E., Merlotti, A., Dall’Olio, D., Sala, C., Pandolfi, S., Remondini, D., Rustici, A., Pastore, L.V., Scarpetti, L., Bortolani, B., Cercenelli, L., Brunocilla, E., Marcelli, E., Coppola, F., Castellani, G.: Effectiveness of radiomic zot features in the automated discrimination of oncocytoma from clear cell renal cancer. Journal of Personalized Medicine **13**(3) (2023) 10.3390/jpm13030478 10.3390/jpm13030478 PMC10052019 36983660

[37] Ferro, M., Cobelli, O., Musi, G., Giudice, F., Carrieri, G., Busetto, G.M., Falagario, U.G., Sciarra, A., Maggi, M., Crocetto, F., Barone, B., Caputo, V.F., Marchioni, M., Lucarelli, G., Imbimbo, C., Mistretta, F.A., Luzzago, S., Vartolomei, M.D., Cormio, L., Autorino, R., Tătaru, O.S.: Radiomics in prostate cancer: An up-to-date review. Therapeutic Advances in Urology **14** (2022) 10.1177/17562872221109020 10.1177/17562872221109020 PMC9260602 35814914

[38] Aftab, K., Aamir, F.B., Mallick, S., Mubarak, F., Pope, W.B., Mikkelsen, T., Rock, J.P., Enam, S.A.: Radiomics for precision medicine in glioblastoma. Journal of neuro-oncology, 1–15 (2022) 10.1007/s11060-021-03933-1 10.1007/s11060-021-03933-1 35020109

[39] Spadarella, G., Perillo, T., Ugga, L., Cuocolo, R.: Radiomics in cardiovascular disease imaging: from pixels to the heart of the problem. Current Cardiovascular Imaging Reports, 1–11 (2022) 10.1007/s12410-022-09563-z

[40] Biondi, R., Renzulli, M., Golfieri, R., Curti, N., Carlini, G., Sala, C., Giampieri, E., Remondini, D., Vara, G., Cattabriga, A., Cocozza, M.A., Pastore, L.V., Brandi, N., Palmeri, A., Scarpetti, L., Tanzarella, G., Cescon, M., Ravaioli, M., Castellani, G., Coppola, F.: Machine learning pipeline for the automated prediction of microvascularinvasion in hepatocellularcarcinomas. Applied Sciences **13**(3) (2023) 10.3390/app13031371

[41] Lei, C., Wei, W., Liu, Z., Xiong, Q., Yang, C., Yang, M., Zhang, L., Zhu, T., Zhuang, X., Liu, C., Liu, Z., Tian, J., Wang, K.: Mammography-based radiomic analysis for predicting benign bi-rads category 4 calcifications. European journal of radiology **121**, 108711 (2019) 10.1016/j.ejrad.2019.108711 10.1016/j.ejrad.2019.108711 31677544

[42] Stelzer, P., Steding, O., Raudner, M., Euller, G., Clauser, P., Baltzer, P.: Combined texture analysis and machine learning in suspicious calcifications detected by mammography: Potential to avoid unnecessary stereotactical biopsies. European Journal of Radiology **132**, 109309 (2020) 10.1016/j.ejrad.2020.109309 10.1016/j.ejrad.2020.109309 33010682

[43] Marathe, K., Marasinou, C., Li, B., Nakhaei, N., Li, B., Elmore, J.G., Shapiro, L., Hsu, W.: Automated quantitative assessment of amorphous calcifications: Towards improved malignancy risk stratification. Computers in Biology and Medicine **146**, 105504 (2022) 10.1016/j.compbiomed.2022.105504 10.1016/j.compbiomed.2022.105504 PMC9839357 35525068

[44] Loizidou K, Skouroumouni G, Nikolaou C, Pitris C (2020). An automated breast micro-calcification detection and classification technique using temporal subtraction of mammograms. IEEE Access.

[45] Fanizzi A, Basile T, Losurdo L, Bellotti R, Bottigli U, Dentamaro R, Didonna V, Fausto A, Massafra R, Moschetta M, Popescu O, Tamborra P, Tangaro S, La Forgia D (2020). A machine learning approach on multiscale texture analysis for breast microcalcification diagnosis. BMC bioinformatics.

[46] Ekpo, E.U., Alakhras, M., Brennan, P.: Errors in mammography cannot be solved through technology alone. Asian Pacific journal of cancer prevention: APJCP **19**(2), 291 (2018). 10.22034/APJCP.2018.19.2.291 10.22034/APJCP.2018.19.2.291 PMC5980911 29479948

[47] Papanikolaou N, Matos C, Koh DM (2020). How to develop a meaningful radiomic signature for clinical use in oncologic patients. Cancer Imaging.

[48] Prinzi, F., Militello, C., Scichilone, N., Gaglio, S., Vitabile, S.: Explainable machine-learning models for covid-19 prognosis prediction using clinical, laboratory and radiomic features. IEEE Access, 11, 121492-121510 (2023) 10.1109/ACCESS.2023.3327808

[49] Wei P (2021). Radiomics, deep learning and early diagnosis in oncology. Emerging topics in life sciences.

[50] Soda, P., D’Amico, N.C., Tessadori, J., Valbusa, G., Guarrasi, V., Bortolotto, C., Akbar, M.U., Sicilia, R., Cordelli, E., Fazzini, D., *et al.*: Aiforcovid: Predicting the clinical outcomes in patients with covid-19 applying ai to chest-x-rays. an italian multicentre study. Medical image analysis **74**, 102216 (2021) 10.1016/j.media.2021.102216 10.1016/j.media.2021.102216 PMC8401374 34492574

[51] Weld DS, Bansal G (2019). The challenge of crafting intelligible intelligence. Communications of the ACM.

[52] Guidotti R, Monreale A, Ruggieri S, Turini F, Giannotti F, Pedreschi D (2018). A survey of methods for explaining black box models. ACM computing surveys (CSUR).

[53] Zwanenburg, A., Vallières, M., Abdalah, M.A., Aerts, H.J.W.L., Andrearczyk, V., Apte, A., Ashrafinia, S., Bakas, S., Beukinga, R.J., Boellaard, R., Bogowicz, M., Boldrini, L., Buvat, I., Cook, G.J.R., Davatzikos, C., Depeursinge, A., Desseroit, M.-C., Dinapoli, N., Dinh, C.V., Echegaray, S., El Naqa, I., Fedorov, A.Y., Gatta, R., Gillies, R.J., Goh, V., Götz, M., Guckenberger, M., Ha, S.M., Hatt, M., Isensee, F., Lambin, P., Leger, S., Leijenaar, R.T.H., Lenkowicz, J., Lippert, F., Losnegård, A., Maier-Hein, K.H., Morin, O., Müller, H., Napel, S., Nioche, C., Orlhac, F., Pati, S., Pfaehler, E.A.G., Rahmim, A., Rao, A.U.K., Scherer, J., Siddique, M.M., Sijtsema, N.M., Socarras Fernandez, J., Spezi, E., Steenbakkers, R.J.H.M., Tanadini-Lang, S., Thorwarth, D., Troost, E.G.C., Upadhaya, T., Valentini, V., Dijk, L.V., Griethuysen, J., Velden, F.H.P., Whybra, P., Richter, C., Löck, S.: The image biomarker standardization initiative: standardized quantitative radiomics for high-throughput image-based phenotyping. Radiology **295**(2), 328–338 (2020) 10.1148/radiol.2020191145 10.1148/radiol.2020191145 PMC7193906 32154773

[54] Van Griethuysen JJ, Fedorov A, Parmar C, Hosny A, Aucoin N, Narayan V, Beets-Tan RG, Fillion-Robin J-C, Pieper S, Aerts HJ (2017). Computational radiomics system to decode the radiographic phenotype. Cancer research.

[55] Lee S-H, Park H, Ko ES (2020). Radiomics in breast imaging from techniques to clinical applications: a review. Korean Journal of Radiology.

[56] Haralick RM, Shanmugam K, Dinstein IH (1973). Textural features for image classification. IEEE Transactions on systems, man, and cybernetics.

[57] Galloway, M.M.: Texture analysis using gray level run lengths. Computer graphics and image processing **4**(2), 172–179 (1975) 10.1016/S0146-664X(75)80008-6 Get rights and content

[58] Chu A, Sehgal CM, Greenleaf JF (1990). Use of gray value distribution of run lengths for texture analysis. Pattern Recognition Letters.

[59] Xu D-H, Kurani AS, Furst JD, Raicu DS (2004). Run-length encoding for volumetric texture. Heart.

[60] Amadasun M, King R (1989). Textural features corresponding to textural properties. IEEE Transactions on systems, man, and Cybernetics.

[61] Thibault, G., FERTIL, B., Navarro, C., Pereira, S., Lévy, N., Sequeira, J., MARI, J.-L.: Texture indexes and gray level size zone matrix application to cell nuclei classification. (2009)

[62] Sun, C., Wee, W.G.: Neighboring gray level dependence matrix for texture classification. Computer Vision, Graphics, and Image Processing **23**(3), 341–352 (1983) 10.1016/0734-189X(83)90032-4

[63] Chalkidou, A., O’Doherty, M.J., Marsden, P.K.: False discovery rates in pet and ct studies with texture features: a systematic review. PloS one **10**(5), 0124165 (2015) 10.1371/journal.pone.0124165 10.1371/journal.pone.0124165 PMC4418696 25938522

[64] Militello C, Prinzi F, Sollami G, Rundo L, La Grutta L, Vitabile S (2023). Ct radiomic features and clinical biomarkers for predicting coronary artery disease. Cognitive Computation.

[65] Oikonomou EK, Williams MC, Kotanidis CP, Desai MY, Marwan M, Antonopoulos AS, Thomas KE, Thomas S, Akoumianakis I, Fan LM, Kesavan S, Herdman L, Alashi A, Centeno EH, Lyasheva M, Griffin BP, Flamm SD, Shirodaria C, Sabharwal N, Kelion A, Dweck MR, Van Beek EJR, Deanfield J, Hopewell JC, Neubauer S, Channon KM, Achenbach S, Newby DE, Antoniades C (2019). A novel machine learning-derived radiotranscriptomic signature of perivascular fat improves cardiac risk prediction using coronary CT angiography. European Heart Journal.

[66] Niu Q, Jiang X, Li Q, Zheng Z, Du H, Wu S, Zhang X (2018). Texture features and pharmacokinetic parameters in differentiating benign and malignant breast lesions by dynamic contrast enhanced magnetic resonance imaging. Oncology Letters.

[67] Raschka, S.: Mlxtend: Providing machine learning and data science utilities and extensions to python’s scientific computing stack. Journal of Open Source Software **3**(24), 638 (2018) 10.21105/joss.00638

[68] Chawla NV, Bowyer KW, Hall LO, Kegelmeyer WP (2002). Smote: Synthetic minority over-sampling technique. Journal of Artificial Intelligence Research.

[69] He, H., Bai, Y., Garcia, E.A., Li, S.: Adasyn: Adaptive synthetic sampling approach for imbalanced learning. In: 2008 IEEE International Joint Conference on Neural Networks (IEEE World Congress on Computational Intelligence), pp. 1322–1328 (2008). 10.1109/IJCNN.2008.4633969

[70] Han, H., Wang, W.-Y., Mao, B.-H.: Borderline-smote: a new over-sampling method in imbalanced data sets learning. In: International Conference on Intelligent Computing, pp. 878–887 (2005). 10.1007/11538059_91. Springer

[71] Last, F., Douzas, G., Bacao, F.: Oversampling for imbalanced learning based on k-means and smote. arxiv 2017. arXiv preprint arXiv:1711.00837 **2**

[72] Mooijman, P., Catal, C., Tekinerdogan, B., Lommen, A., Blokland, M.: The effects of data balancing approaches: A case study. Applied Soft Computing **132**, 109853 (2023) 10.1016/j.asoc.2022.109853

[73] Kovács, G.: An empirical comparison and evaluation of minority oversampling techniques on a large number of imbalanced datasets. Applied Soft Computing **83**, 105662 (2019) 10.1016/j.asoc.2019.105662

[74] Azhar, N.A., Pozi, M.S.M., Din, A.M., Jatowt, A.: An investigation of smote based methods for imbalanced datasets with data complexity analysis. IEEE Transactions on Knowledge and Data Engineering (2022) 10.1109/TKDE.2022.3179381

[75] Kabiraj, S., Raihan, M., Alvi, N., Afrin, M., Akter, L., Sohagi, S.A., Podder, E.: Breast cancer risk prediction using xgboost and random forest algorithm. In: 2020 11th International Conference on Computing, Communication and Networking Technologies (ICCCNT), pp. 1–4 (2020). 10.1109/ICCCNT49239.2020.9225451. IEEE

[76] Ghiasi, M.M., Zendehboudi, S.: Application of decision tree-based ensemble learning in the classification of breast cancer. Computers in Biology and Medicine **128**, 104089 (2021) 10.1016/j.compbiomed.2020.104089 10.1016/j.compbiomed.2020.104089 33338982

[77] Kotsiantis SB (2013). Decision trees: a recent overview. Artificial Intelligence Review.

[78] Prinzi, F., Orlando, A., Gaglio, S., Midiri, M., Vitabile, S.: Ml-based radiomics analysis for breast cancer classification in dce-mri. In: Mahmud, M., Ieracitano, C., Kaiser, M.S., Mammone, N., Morabito, F.C. (eds.) Applied Intelligence and Informatics, pp. 144–158. Springer, Cham (2022). 10.1007/978-3-031-24801-6_11

[79] Martel, A.L.: Cad and machine learning for breast mri. Breast MRI for High-risk Screening, 97–111 (2020) 10.1007/978-3-030-41207-4_7

[80] Dong, T., Yang, C., Cui, B., Zhang, T., Sun, X., Song, K., Wang, L., Kong, B., Yang, X.: Development and validation of a deep learning radiomics model predicting lymph node status in operable cervical cancer. Frontiers in Oncology **10**, 464 (2020) 10.3389/fonc.2020.00464 10.3389/fonc.2020.00464 PMC7179686 32373511

[81] Liu M, Mao N, Ma H, Dong J, Zhang K, Che K, Duan S, Zhang X, Shi Y, Xie H (2020). Pharmacokinetic parameters and radiomics model based on dynamic contrast enhanced mri for the preoperative prediction of sentinel lymph node metastasis in breast cancer. Cancer Imaging.

[82] Zhou J, Zhang Y, Chang K-T, Lee KE, Wang O, Li J, Lin Y, Pan Z, Chang P, Chow D (2020). Diagnosis of benign and malignant breast lesions on dce-mri by using radiomics and deep learning with consideration of peritumor tissue. Journal of Magnetic Resonance Imaging.

[83] Nam, K.J., Park, H., Ko, E.S., Lim, Y., Cho, H.-H., Lee, J.E.: Radiomics signature on 3t dynamic contrast-enhanced magnetic resonance imaging for estrogen receptor-positive invasive breast cancers: Preliminary results for correlation with oncotype dx recurrence scores. Medicine **98**(23) (2019) 10.1097/MD.0000000000015871 10.1097/MD.0000000000015871 PMC6571434 31169691

[84] Fan, M., Li, H., Wang, S., Zheng, B., Zhang, J., Li, L.: Radiomic analysis reveals dce-mri features for prediction of molecular subtypes of breast cancer. PloS one **12**(2), 0171683 (2017) 10.1371/journal.pone.0171683 10.1371/journal.pone.0171683 PMC5293281 28166261

[85] Junior JRF, Koenigkam-Santos M, Cipriano FEG, Fabro AT, Azevedo-Marques PM (2018). Radiomics-based features for pattern recognition of lung cancer histopathology and metastases. Computer methods and programs in biomedicine.

[86] Di Stefano, V., Prinzi, F., Luigetti, M., Russo, M., Tozza, S., Alonge, P., Romano, A., Sciarrone, M.A., Vitali, F., Mazzeo, A., Gentile, L., Palumbo, G., Manganelli, F., Vitabile, S., Brighina, F.: Machine learning for early diagnosis of attrv amyloidosis in non-endemic areas: A multicenter study from italy. Brain Sciences **13**(5), 805 (2023) 10.3390/brainsci13050805 10.3390/brainsci13050805 PMC10216819 37239276

[87] ELI5 Website: Eli5 Documentation. (Last accessed 31-Mar-2022) (2022). https://eli5.readthedocs.io/en/latest/index.html

[88] Huang S-Y, Franc BL, Harnish RJ, Liu G, Mitra D, Copeland TP, Arasu VA, Kornak J, Jones EF, Behr SC, Hylton NM, Price ER, Esserman L, Youngho S (2018). Exploration of pet and mri radiomic features for decoding breast cancer phenotypes and prognosis. NPJ breast cancer.

[89] Parekh VS, Jacobs MA (2017). Integrated radiomic framework for breast cancer and tumor biology using advanced machine learning and multiparametric mri. NPJ breast cancer.

[90] Parekh VS, Jacobs MA (2020). Multiparametric radiomics methods for breast cancer tissue characterization using radiological imaging. Breast cancer research and treatment.

[91] Liu, J., Sun, D., Chen, L., Fang, Z., Song, W., Guo, D., Ni, T., Liu, C., Feng, L., Xia, Y., Zhang, X., Li, C.: Radiomics analysis of dynamic contrast-enhanced magnetic resonance imaging for the prediction of sentinel lymph node metastasis in breast cancer. Frontiers in Oncology **9**, 980 (2019) 10.3389/fonc.2019.00980 10.3389/fonc.2019.00980 PMC6778833 31632912

[92] Li M, Zhu L, Zhou G, He J, Jiang Y, Chen Y (2021). Predicting the pathological status of mammographic microcalcifications through a radiomics approach. Intelligent Medicine.

[93] Li J, Song Y, Xu S, Wang J, Huang H, Ma W, Jiang X, Wu Y, Cai H, Li L (2019). Predicting underestimation of ductal carcinoma in situ: a comparison between radiomics and conventional approaches. International journal of computer assisted radiology and surgery.

[94] Ramos-Pollán R, Guevara-López MA, Suárez-Ortega C, Díaz-Herrero G, Franco-Valiente JM, Rubio-del-Solar M, González-de-Posada N, Vaz MAP, Loureiro J, Ramos I (2012). Discovering mammography-based machine learning classifiers for breast cancer diagnosis. Journal of medical systems.

[95] Caroprese, L., Vocaturo, E., Zumpano, E.: Argumentation approaches for explanaible ai in medical informatics. Intelligent Systems with Applications **16**, 200109 (2022) 10.1016/j.iswa.2022.200109

[96] Kumaraswamy, E., Kumar, S., Sharma, M.: An invasive ductal carcinomas breast cancer grade classification using an ensemble of convolutional neural networks. Diagnostics **13**(11), 1977 (2023) 10.3390/diagnostics13111977 10.3390/diagnostics13111977 PMC10252802 37296828

[97] Kumar S, Sharma S (2022). Sub-classification of invasive and non-invasive cancer from magnification independent histopathological images using hybrid neural networks. Evolutionary Intelligence.

[98] Sharma S, Mehra R, Kumar S (2021). Optimised cnn in conjunction with efficient pooling strategy for the multi-classification of breast cancer. IET Image Processing.

[99] Veer SN, Riste L, Cheraghi-Sohi S, Phipps DL, Tully MP, Bozentko K, Atwood S, Hubbard A, Wiper C, Oswald M (2021). Trading off accuracy and explainability in ai decision-making: findings from 2 citizens’ juries. Journal of the American Medical Informatics Association.

[100] Bell, A., Solano-Kamaiko, I., Nov, O., Stoyanovich, J.: It’s just not that simple: an empirical study of the accuracy-explainability trade-off in machine learning for public policy. In: Proceedings of the 2022 ACM Conference on Fairness, Accountability, and Transparency, pp. 248–266 (2022). 10.1145/3531146.3533090

