# Supplementary material for: Interpretable Radiomic Signature for Breast Microcalcification Detection and Classification
Source: J Imaging Inform Med. 2024 Feb 13;37(3):1038–53. doi: 10.1007/s10278-024-01012-1 (PMC11169144; doi:10.1007/s10278-024-01012-1)
Supplement: Supplementary file 1 — (pdf 134 KB) [file 10278_2024_1012_MOESM1_ESM.pdf]

Supplementary Material

Radiomic Features

In our study, we conformed to the standardization process in line with the Imaging Biomarker Standardization Initiative (IBSI) ***[Zwanenburg et al.]*** to ensure that the extracted features adhered to the required standards. To achieve this, the PyRadiomics library was used (version 3.0.1) ***[Van Griethuysen et al.]***, which is designed to be fully IBSI compliant.

The extracted features and their mathematical formulation were obtained from PyRadiomics and are comprehensively documented in its documentation (https://pyradiomics.readthedocs.io/en/v3.0.1/features.html#). This ensures that the data and methodologies are accessible and transparent for readers interested in further examining our analyses.

In this work the following categories of features were extracted:

- First Order
- Gray Level Cooccurrence Matrix (GLCM)
- Gray Level Run Length Matrix (GLRLM)
- Gray Level Size Zone Matrix (GLSZM)
- Neighboring Gray Tone Difference Matrix (NGTDM)
- Gray Level Dependence Matrix (GLDM)

The tables below provide a brief description of the categories, list the features extracted and finally provide some examples.

References

Zwanenburg, A., Vallières, M., Abdalah, M. A., Aerts, H. J., Andrearczyk, V., Apte, A., ... & Löck, S. (2020). The image biomarker standardization initiative: standardized quantitative radiomics for high-throughput image-based phenotyping. Radiology, 295(2), 328-338.

Van Griethuysen, J. J., Fedorov, A., Parmar, C., Hosny, A., Aucoin, N., Narayan, V., ... & Aerts, H. J. (2017). Computational radiomics system to decode the radiographic phenotype. Cancer research, 77(21), e104-e107.

Table 1 - First order features

Definition	Features	Examples
First-order statistics describe the distribution of voxel intensities within the image region defined by the mask through commonly used and basic metrics.	1. Energy<br>2. TotalEnergy<br>3. Entropy<br>4. Minimum<br>5. 10Percentile<br>6. 90Percentile<br>7. Maximum<br>8. Mean<br>9. Median<br>10. InterquartileRange<br>11. Range<br>12. MeanAbsoluteDeviation<br>13. RobustMeanAbsoluteDeviation<br>14. RootMeanSquared<br>15. Skewness<br>16. Kurtosis<br>17. Variance<br>18. Uniformity	**Energy:** is a measure of the magnitude of voxel values in an image. A larger value implies a greater sum of the squares of these values.<br><br>**Skewness:** measures the asymmetry of the distribution of values about the Mean value. Depending on where the tail is elongated and the mass of the distribution is concentrated, this value can be positive or negative.

Table 2 - Gray Level Co-occurrence Matrix (GLCM) features

**Definition**	**Features**	**Examples**
A Gray Level Co-occurrence Matrix (GLCM) of size $N_g \times N_g$ describes the second-order joint probability function of an image region constrained by the mask. The $(i, j)^{th}$ element of this matrix represents the number of times the combination of levels I and j occur in two pixels in the image, that are separated by a distance of δ pixels along angle $\boldsymbol{\theta}$.	1. Autocorrelation<br>2. JointAverage<br>3. ClusterProminence<br>4. ClusterShade<br>5. ClusterTendency<br>6. Contrast<br>7. Correlation<br>8. DifferenceAverage<br>9. DifferenceEntropy<br>10. DifferenceVariance<br>11. JointEnergy<br>12. JointEntropy<br>13. InformationalMeasureofCorrelation1<br>14. InformationalMeasureofCorrelation2<br>15. InverseDifferenceMoment<br>16. MaximumCorrelationCoefficient<br>17. InverseDifferenceMomentNormalized<br>18. InverseDifference<br>19. InverseDifferenceNormalized<br>20. InverseVariance<br>21. MaximumProbability<br>22. SumAverage<br>23. SumEntropy<br>24. SumSquares	**Autocorrelation**: is a measure of the magnitude of the fineness and coarseness of texture.<br><br>**Contrast**: is a measure of the local intensity variation. A larger value correlates with a greater disparity in intensity values among neighboring voxels.

Table 3 - Gray Level Size Zone (GLSZM) features

Definition	Features	Examples
A Gray Level Size Zone (GLSZM) quantifies gray level zones in an image. A gray level zone is defined as a the number of connected voxels that share the same gray level intensity.	1. SmallAreaEmphasis<br>2. LargeAreaEmphasis<br>3. GrayLevelNonUniformity<br>4. GrayLevelNonUniformityNormalized<br>5. SizeZoneNonUniformity<br>6. SizeZoneNonUniformityNormalized<br>7. ZonePercentage<br>8. GrayLevelVariance<br>9. ZoneVariance<br>10. ZoneEntropy<br>11. LowGrayLevelZoneEmphasis<br>12. SmallAreaLowGrayLevelEmphasis<br>13. SmallAreaHighGrayLevelEmphasis<br>14. HighGrayLevelZoneEmphasis<br>15. LargeAreaLowGrayLevelEmphasis<br>16. LargeAreaHighGrayLevelEmphasis	**Low Gray Level Zone Emphasis:** measures the distribution of lower gray-level size zones, with a higher value indicating a greater proportion of lower gray-level values and size zones in the image.<br><br>**Large Area High Gray Level Emphasis**: measures the proportion in the image of the joint distribution of larger size zones with higher gray-level values.

Table 4 - Gray Level Run Length Matrix (GLRLM) features

Definition	Features	Examples
A Gray Level Run Length Matrix (GLRLM) quantifies gray level runs, which are defined as the length in number of pixels, of consecutive pixels that have the same gray level value.	1. ShortRunEmphasis<br>2. LongRunEmphasis<br>3. GrayLevelNonUniformity<br>4. GrayLevelNonUniformityNormalized<br>5. RunLengthNonUniformity<br>6. RunLengthNonUniformityNormalized<br>7. RunPercentage<br>8. GrayLevelVariance<br>9. RunVariance<br>10. RunEntropy<br>11. LowGrayLevelRunEmphasis<br>12. HighGrayLevelRunEmphasis<br>13. ShortRunLowGrayLevelEmphasis<br>14. ShortRunHighGrayLevelEmphasis<br>15. LongRunLowGrayLevelEmphasis<br>16. LongRunHighGrayLevelEmphasis	**Long Run Emphasis:** is a measure of the distribution of long run lengths, with a greater value indicative of longer run lengths and more coarse structural textures.<br><br>**Run Entropy**: measures the uncertainty/randomness in the distribution of run lengths and gray levels. A higher value indicates more heterogeneity in the texture patterns.

Table 5 - Neighbouring Gray Tone Difference Matrix (NGTDM) features

Definition	Features	Example
A Neighbouring Gray Tone Difference Matrix quantifies the difference between a gray value and the average gray value of its neighbours within distance δ.	1. Coarseness<br>2. Contrast<br>3. Busyness<br>4. Complexity<br>5. Strength	**Coarseness:** is a measure of average difference between the center voxel and its neighborhood and is an indication of the spatial rate of change. A higher value indicates a lower spatial change rate and a locally more uniform texture.<br><br>**Complexity**: an image is considered complex when there are many primitive components in the image, i.e. the image is non-uniform and there are many rapid changes in gray level intensity.

Table 6 - Gray Level Dependence Matrix (GLDM) features

Definition	Features	Example
A Gray Level Dependence Matrix (GLDM) quantifies gray level dependencies in an image. A gray level dependency is defined as the number of connected voxels within distance δ that are dependent on the center voxel.	1. SmallDependenceEmphasis<br>2. LargeDependenceEmphasis<br>3. GrayLevelNonUniformity<br>4. DependenceNonUniformity<br>5. DependenceNonUniformityNormalized<br>6. GrayLevelVariance<br>7. DependenceVariance<br>8. DependenceEntropy<br>9. LowGrayLevelEmphasis<br>10. HighGrayLevelEmphasis<br>11. SmallDependenceLowGrayLevelEmphasis<br>12. SmallDependenceHighGrayLevelEmphasis<br>13. LargeDependenceLowGrayLevelEmphasis<br>14. LargeDependenceHighGrayLevelEmphasis	**Low Gray Level Emphasis:** Measures the distribution of low gray-level values, with a higher value indicating a greater concentration of low gray-level values in the image.<br><br>**Large Dependence Emphasis**: A measure of the distribution of large dependencies, with a greater value indicative of larger dependence and more homogeneous textures.

Leave-One-Out performance

Table 7 - Detection performance computed in Leave-One-Out cv.

Model	Accuracy	AUROC	Specificity	Sensitivity	PPV	NPV
XGB	0.799	0.881	0.831	0.766	0.820	0.779
SVM	0.772	0.847	0.794	0.750	0.786	0.759
RF	0.793	0.870	0.824	0.763	0.814	0.775

Table 8 - Classification performance computed in Leave-One-Out cv.

Model	Accuracy	AUROC	Specificity	Sensitivity	PPV	NPV
XGB	0.889	0.947	0.878	0.898	0.881	0.896
SVM	0.853	0.907	0.898	0.808	0.889	0.824
RF	0.886	0.943	0.893	0.879	0.892	0.880
